# Revealing Hidden Cognitive Language Patterns in Brain Injury: Can Modifiers and Function Words Play a Role in Neuroplasticity?

**DOI:** 10.3390/brainsci15111239

**Published:** 2025-11-19

**Authors:** Marisol Roldán-Palacios, Aurelio López-López

**Affiliations:** Instituto Nacional de Astrofísica Óptica y Electrónica, Santa María Tonantzintla, Puebla CP 72840, Mexico; marppalacios@inaoep.mx

**Keywords:** traumatic brain injury (TBI), brain plasticity, narrative discourse analysis, linguistic feature evaluation, statistical learning, therapeutics

## Abstract

**Background:** Although modifiers and function words are critical in cognitive linguistic assessments and cognitive training has proven to promote synaptic neural activity, they often receive limited attention, particularly in computational data-scarce settings. This study addresses communication difficulties associated with cognitive impairments using engineering data, a design to improve the evaluation of language attributes, applied specifically to these elements. A framework was developed to analyze potential language alterations resulting from traumatic brain injury (tbi), using narrative samples, primary data, and unconventional methods to overcome the limitations of existing resources. **Methods:** The core technique involves pairing language attributes based on defined relationships and assessing responses using standard statistical learning methods. Direct and normalized evaluations of variables, calculated using the Northwestern Narrative Language Analysis (nnla) profile from the original data, serve as benchmarks. The Area Under the Curve (auc) metric with the corresponding statistical support are reported. **Results:** The results indicate that the proposed method revealed informative patterns involving modifiers and function words that remained hidden in the baseline approaches. Although some exceptions were observed, results showed a substantially consistent behavior, and the responses achieved promote their use in a clinical setting. **Conclusions:** The findings can provide valuable directions for theoretical and applied research in language assessment. Identifying specific points of breakdown within language structures can improve the accuracy of rehabilitation plans and better leverage the neuroplastic response of the brain for recovery.

## 1. Introduction

Language engages extensive regions of the cerebral cortex and midbrain [1,2,3]. In other words, humans rely heavily on widespread brain areas to produce and comprehend language, making language dysfunction a common consequence of brain trauma. This analysis focuses specifically on the subtle effects of severe [4] traumatic brain injury (TBI) on language and, more importantly, on how to identify disrupted item-based relationships within the language system. Understanding these disruptions can reveal critical aspects to consider in developing effective interventions for language impairments.

This study addresses acquired language deficits associated with cognitive impairment through computational methods. Rather than adopting a general “big data” approach  [5], it proposes a focused framework to examine “one of the most important products of human cerebral action” [6]—language—that is “full of neat systems on the one hand and odd quirks on the other” [7]. This perspective grounds the study in both scientific and humanistic frameworks, recognizing language as a uniquely structured yet irregular cognitive function.

Several factors can compromise healthy language functioning, with consequences varying according to the underlying causes, situational conditions, and individual-specific contexts [8,9,10]. Essentially, language disorders can arise from impairments in the attentional, memory, linguistic, or executive cognitive processes that underpin language integration.

In traumatic brain injury (TBI) [11], language deficits are directly linked to the complexity of cognitive-communication disorder [12,13]—also referred to as cognitive-linguistic deficit [14]. Head trauma can lead to distinctive speech patterns, language disorders, and life disruption that warrant focused clinical attention [15,16].

To address the underlying causes of this condition, continuous analysis of discourse-cognition dependencies [17,18] is encouraged, as research has shown that discourse—at various levels—is closely linked to multiple cognitive functions. Therefore, relevant attributes should guide the analysis.

Previous studies [19,20] have underlined the informative potential of adjectives and adverbs. These basic types of modifiers are analyzed alongside function words—such as determiners, prepositions, and conjunctions—which have been theorized to be influenced by cognitive processes [14,21].

However, several concerns have been raised regarding the computational mechanisms [22,23,24,25], particularly emphasizing the biases introduced through engineering processes and their associated risks. Uncertainty—an essential factor in evaluating the reliability of engineering data—is central to these concerns. Therefore, the proposed strategy must be grounded in rational principles, which is the central aim of the present study.

In computational approaches, trimming adjectives and prepositions is part of a text simplification process designed to enhance readability for individuals with limited language comprehension, including children and groups with conditions such as autism or Down syndrome [26]. In pediatric TBI cases, modifiers, verbs, and their complements expanded the knowledge base for the Case-Based Reasoning (CBR) process in AI. This process involves linking new cases with previously resolved ones and reusing the procedures applied in similar situations [27].

Intending a synthetic emulation of agrammatic deficiencies, adjectives, adverbs, and other attributes [28] were removed from grammatically correct sentences. Removing function words, such as conjunctions and prepositions, is a common practice, assuming poor informational content for these attributes [29]. So, modification is often not the primary study focus, and function words are frequently overlooked.

The current understanding of cerebral plasticity in adults with chronic traumatic brain injury (tbi) was reviewed, with a particular focus on the impact of cognitive interventions, given that cognitive impairments are among the most significant functional deficits in this population [30]. The review encompassed a variety of study designs, including group studies, pilot studies, single-case reports, meta-analyses, and systematic reviews. Although many studies were limited by small sample sizes, the absence of control groups, and heterogeneity in both neural and clinical outcomes, general evidence consistently suggests that cognitive interventions can lead to measurable improvements in cognitive functioning.

Improvements in communicative abilities have been observed following cognitive training, and these gains are often accompanied by increased cerebral activity in brain regions associated with pragmatic functioning. Although cognitive-communication rehabilitation remains an emerging field [31], a growing body of evidence supports its critical role in the recovery process. However, several challenges persist, including the high variability and diagnostic ambiguity characteristic of tbi cases, as well as limited clarity in preclinical research, particularly regarding the development and refinement of effective intervention strategies [32].

Cognitive narratology has long guided research on storytelling and the mind from its inception, with recent inquiries exploring “how a focus on the mind-narrative nexus might illuminate the structure and functions of situated storytelling acts” [33]. These investigations aim to explain, among other things, why a given text may possess a particular structure. Despite the recognized importance of narrative skills for everyday communication, they are increasingly overlooked in intervention plans for individuals with cognitive-communication disorders [13].

## 2. Materials and Methods

### 2.1. Framework

The central focus of this study is language, specifically examining aspects of communication that are impaired or disrupted as a result of cranial trauma. The scope is limited to language functions linked to cognition, with particular attention to cases of traumatic brain injury (tbi). The analysis concentrates on the acute phase of the post-recovery period.

All factors relevant to these circumstances are considered essential, including insights from related research fields, as they can significantly influence outcomes and guide critical decision making. Accordingly, this study explores a range of contributing factors, drawing on expert knowledge from disciplines where relevant connections have been identified, as outlined below.

#### 2.1.1. A Narrative Discourse Task

Several functional-cognitive frameworks have been developed to explain language. Regardless of whether these perspectives emphasize cognitive or communicative aspects, they share key assumptions—most notably, the view that the communicative role of language [34] is fundamental to understanding why language systems take the forms they do.

In this context, research has demonstrated significant relationships between discourse and various cognitive mechanisms operating at multiple levels. As noted by  [35], “discourse can be thought of as the mechanism underlying the organization of speech into a coherent flow.” Analyzing discourse provides insight into a range of cognitive processes [36,37]. Different aspects of discourse structure impose distinct demands on the speaker’s language system, enabling access to inferencing and cognitive planning processes involved in producing discourse of varying complexity.

Specifically, examining this distinct aspect of discourse enables access to various cognitive mechanisms [38,39]. In fact, the narrative form is regarded as “a fundamental structure of human cognition” [40], positioning storytelling as closely aligned with processes of understanding. Moreover, both telling and retelling tasks have been found to rely heavily on cognitive processes [41].

**Figure 1:**
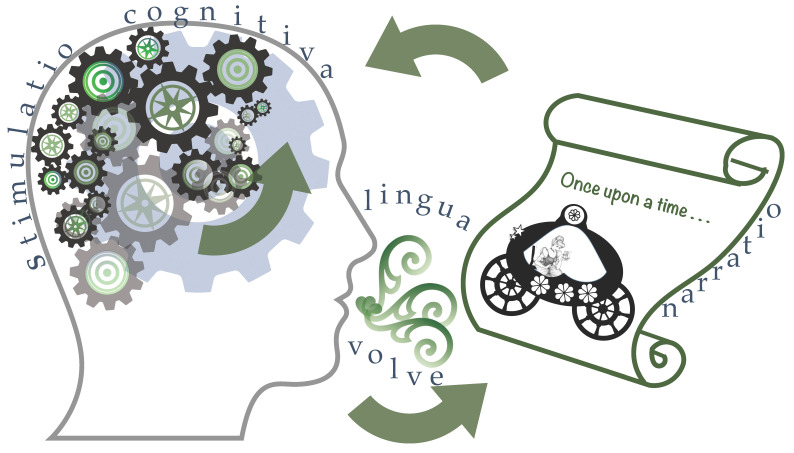
Distinctive integrative functions of the cerebral cortex are reflected in the language mechanism: [6]. Predicated on this symbiotic relationship, more accurate stimulation of affected language could promote neuron connection.

Figure 1 illustrates previously discussed questions regarding the language mechanism and the self-generating language–cognition process involved in constructing narrative discourse. It also highlights the cognitive functions required at various levels for organizing the retelling of a folktale.

Established findings indicate that individuals with moderate to severe tbi often experience difficulties with spoken narrative discourse [13]. As such, narrative discourse analysis may serve as a valuable tool for assessing various aspects of language and cognition [42,43].

#### 2.1.2. Attribute Determination

In narrative discourse, the generation process is associated with storytelling, while story retelling involves exposure to a previously presented narrative. Both forms—whether factual or fictional—share overlapping properties and engage key cognitive functions such as knowledge, memory, abstraction, and reasoning [41,44,45,46].

Each element of discourse structure places distinct demands on the speaker’s linguistic system, facilitating access to inferencing and cognitive planning during discourse production of varying complexity. Therefore, analyzing these elements provides valuable insight into underlying cognitive processes. In other words, examining the different facets of discourse enables the exploration of multiple cognitive mechanisms [39].

Specifically, modification operates alongside predication and reference as one of the highest-level language functions, particularly within the framework of Functional Discourse Grammar [47]. Moreover, modification serves a pragmatic purpose by directing information toward the addressee. As noted, “modifiers connect ideas while adding information” [48]. Even in their simplest forms—such as adjectives and adverbs [49]—qualifying modifications play a crucial role in conducting functional analyses of language [47].

Formally, function words are grammatical elements that indicate structural relationships between words, serving as the thread that holds sentences together [50]. Although they typically carry little or no lexical meaning on their own, words such as although, yet, but, and then exemplify this category. Function words form the supportive framework of coherent text, establishing logical connections between related propositions [19].

In summary, modifiers, function words, and narrative discourse—particularly in the form of storytelling and story-retelling tasks—were identified as key elements in this study, noticed since the setting and these attributes directly influence the results [22].

### 2.2. Problem Formulation

Building on the previous discussion of key aspects and acknowledging the emerging needs within this context, several additional yet fundamental perspectives deserve attention. A primary concern lies in the challenges related to healthcare access. A significant portion of the population lacks access to even basic health services, which in turn makes it particularly difficult to obtain diagnoses that depend on specialized medical equipment.

When examining the intricate aspects of certain health conditions, a key limitation of synthetic data lies in their reliance on incomplete guidelines derived from the current, and often limited, state of knowledge. As a result, the inclusion of original, real-world data becomes critical for ensuring the validity and depth of such studies. These limitations collectively point to the need for exploring alternative approaches—specifically, more accessible methods for indirectly, yet effectively, observing disorders in language cognition.

At the same time, these challenges contribute to the following broader issue: the practical absence of assessments that account for presented contextual factors, particularly in capturing subtle language changes in individuals following tbi. Addressing these gaps through focused research is essential to advance our understanding of the complex phenomena of language disturbance and its interplay with cognitive and other neurological processes.

#### Problem

The central problem involves uncovering how language is disrupted within cognition-related communication processes by analyzing language behavior—specifically, through samples collected from individuals following traumatic brain injury, diagnosed as severe according to the Glasgow Coma Scale [51]—while avoiding the use of synthetic data and limiting the analysis to available, real-world datasets.

### 2.3. Solution Approach

This study focuses on exploring the underutilized potential of specific linguistic attributes in an area where computational research on cognition-related communication disorders remains limited, and the results of the examined attributes can contribute to a more nuanced understanding of such disorders.

To address the research problem, we outline the instruments and methods employed. Language serves as the most direct means of probing potential cognitive impairments, particularly in communication. Given the strong cognitive dependence of modifiers and function words, we analyze their behavior through the following three sets of attribute pairings: two involving combinations of modifiers and function words, and one consisting of pairings between function words alone.

### 2.4. Applied Method

The used technique is guided by a common-sense approach like thinking, perception, and representational models. So, we drew upon principles from several research fields. Principles that were employed to define relationships between pairs of linguistic attributes enabled us to trace their relational dynamics—specifically, to observe how these connections evolve or fluctuate over time.

The relational measures between pairs of attributes—referred to here as proximity—constitute the feature space evaluated using the following two statistical learning methods: Naïve Bayes and Simple Logistic. The models are applied to the raw data that refer to the extracted variables [52] coming from the Northwestern Narrative Analysis [53] profile. Variables evaluated in two distinct forms serve as the basis for comparison. These are termed direct and normalized evaluations as follows: in the direct approach, the data are analyzed in their original form, whereas in the normalized approach, the data are first standardized prior to analysis. Comprehensive technical details can be found in [54].

### 2.5. Underpinnings

**Figure 2:**
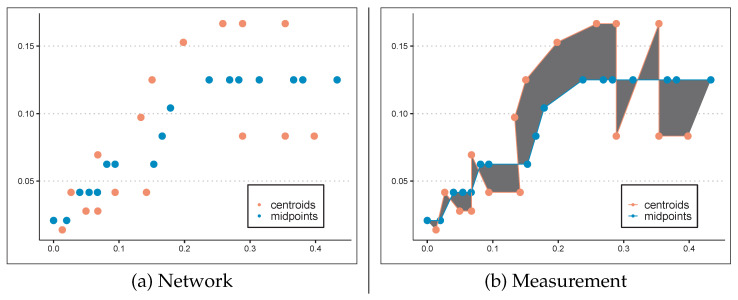
(**a**) The network base corresponding to the pair of attributes (det.-compl.) where (**b**) the proximity measure (gray area) is computed. Time and values of attributes correspond to X-axis and Y-axis values, respectively.

The core measurement in our method is grounded in the principles of logical development and the internal connectivity of components, in a similar vein, in which individual components of a unit gain meaning through their structure, organization, and interconnections which collectively provide logical coherence. Proximity is conceptualized as a unified network that encapsulates pairs of trajectories, each condensing the relationship between attribute pairs over a span of time. Figure 2 illustrates the proximity associated with related attribute pairs (e.g., determiners and complementizers).

Consider narrative construction processes—such as retelling or composing a new story—to illustrate how the language system aligns with the described model [55,56]. Narrative elements are typically introduced in temporal order, likely following specific relational patterns [19]. The duration of narrative development defines a time frame, an abstraction analogous to those found in neuropsychological studies [43]. In the context of language samples, the organization of words shapes the resulting structure, specifically through the identifiable connections among words that constitute the modeled relationships.

In this context, we can identify cooperative elements from the following two complementary approaches: the functional action of language in accomplishing tasks during discourse analysis, and the concept of dependency distance—how syntactic structure and word order influence processing complexity [34]. This is succinctly captured by the notion that “in the process by which language becomes the motor expression of thoughts, the speaker unconsciously adheres to certain laws of word distribution and balance” [20].

To analyze variation in narrative discourse, we examine the proximity relationships between pairs of linguistic attributes—specifically, modifiers versus function words and function words versus other function words. The goal is to show how the use of modifiers and function words becomes disrupted during communication, particularly in relation to cognitive disorders such as those associated with tbi.

### 2.6. Corpus

tbibank is a publicly accessible corpus developed through a standardized process designed to collect language samples for analyzing the altered language patterns resulting from traumatic brain injury tbi [57,58,59]. This data source was developed through collaboration among multiple specialists who discussed, defined, and outlined an international standard protocol aimed at capturing meaningful information about the study’s subject [59]. Specialists followed established protocols to minimize subjective practices during data preparation [60] while also ensuring compliance with principles of ecological validity [61].

The corpus comprises a range of monologic narrative discourse tasks, as discourse production engages cognitive, linguistic, and social abilities [13,59,62]. These tasks include both retelling and generative narratives, which are recognized as innovative discourse measures [63]. All transcripts were standardized according to a defined format to facilitate the extraction of specific indices for language analysis. Additionally, the transcripts were validated using statistical tests, demonstrating a coincidence rate exceeding 90% [11].

### 2.7. Experimental Setting

Table 1 presents the set of attributes in detail. First observe that determiners (det.) contain det:articles and det:demonstratives, and pronouns (pro.) include possessive determiners but exclude ‘wh’ interrogatives and relative pronouns. Conjunctions (conj.) include coordinators but disregard ‘wh’ conjunctions, and modals include modals and modal auxiliaries. Notice that the **#** symbol stands for number (e.g., # of adverbs means number of adverbs).

The pairs of attributes and their respective number of instances per group are detailed in Table 2. Note that the pair (compl., modals) is the only one significantly unbalanced; the other pairs remain relatively well balanced—an important observation for the interpretation of results.

Functions and indices are identified as the primary units through which narratives can be evaluated—either as predominantly functional or indicial. Popular tales [55] serve as examples of functional narratives, while psychological novels illustrate the indicial type. In this study, the narrative tasks varied between the groups as follows: the study group was asked to retell the Cinderella story, while the control group generated a story based on a picture prompt. Both tasks were designed to include a clear beginning, middle, and end [60]. Both story generation and the retelling tasks are highly dependent on cognitive processing [41].

Observe that the method relies on a basic story structure, as described in [65], consisting of a setting—main characters, and a time-and-space context—and a series of episodes. These episodes incorporate elements such as events, reactions, goals, attempts, consequences, and solutions, which can be connected additively (and), temporally (then), or causally (because). Our focus is not on the precision of the stories in terms of specific tasks, but rather on the connections between the elements that compose the narratives.

## 3. Results

The trajectory behavior was analyzed using a proximity-based technique. The resulting data were then evaluated with two statistical methods—Naïve Bayes and Simple Logistic. To reduce bias, a 10-fold cross-validation technique [66] was applied. For evaluation, the area under the roc curve (auc) [67] was used, as a widely accepted metric in clinical studies.

**Figure 3:**
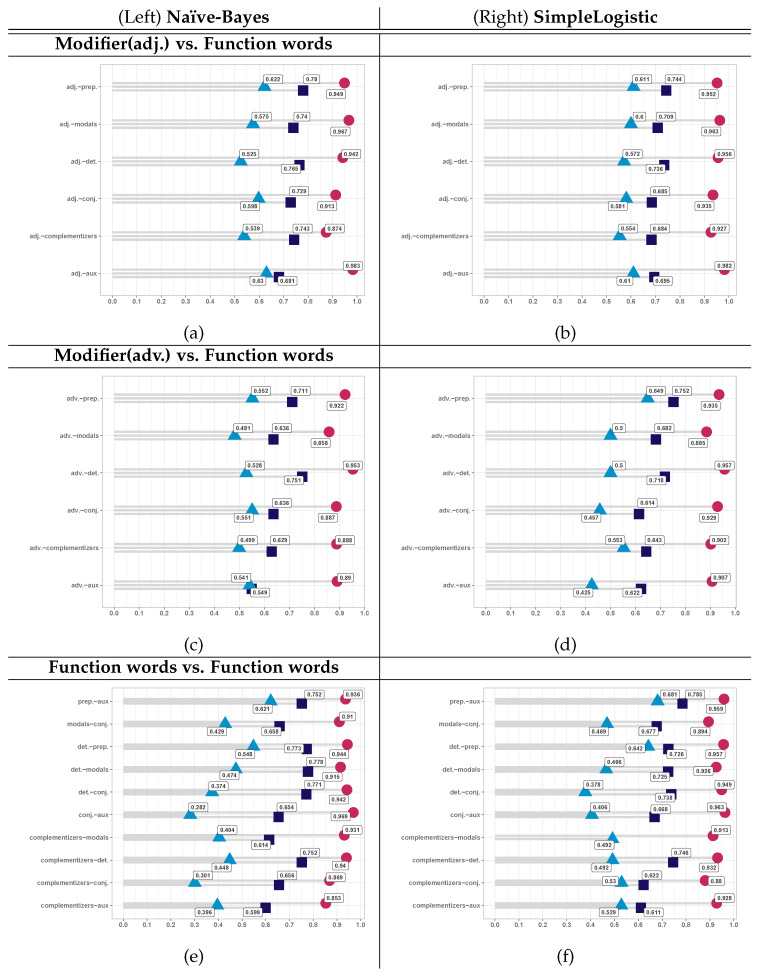
AUC evaluations for attribute pairs. It shows the results for proximity and basis at the 3-month stage. Each symbol corresponds to the measure at hand, [navy blue] square (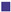) for direct base, [blue] triangle (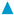) for the normalized base, and [pink] circle (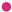) for proximity. Parts (**a**,**c**,**e**) on the left corresponds to NaïveBayes and parts (**b**,**d**,**f**) to SimpleLogistic responses. Combinations of adj-Function words, adv-Function words, and Function words-Function words are in the first, second and third row in that order.

Graphically, Figure 3a,b show the combinations of adjectives with function words, while Figure 3c,d illustrate the combinations of adverbs with function words. Figure 3e,f display the results for pairs of function words. In each case, the left graph corresponds to the Naïve Bayes method, and the right graph corresponds to the Simple Logistic method.

The data in the graphs are organized as follows: the vertical axis lists the attribute combinations, with each pair including three evaluations—direct raw data, normalized raw data, and the proximity measure—represented in blue, yellow, and red, respectively. The horizontal axis shows the auc values. Evaluations based on raw data serve as the basis for comparison. Note that the data in the graphs are arranged in ascending order based on the outcomes of the normalized raw data. This arrangement highlights the gap between proximity-based evaluations and baseline results.

The same data are also presented in the accompanying tables, which include the 95% confidence intervals (ci). The standard error, confidence intervals, and sample sizes used to substantiate statistical power were calculated based on the methodology of Hanley & McNeil [68]. Values in gray do not meet the minimum sample size required for sensitivity analysis. Underlined values meet the minimum sample size necessary to achieve 80% ci of statistical power. The values that are neither grayed nor underlined meet the minimum sample size for 90% power and, in most cases, also fulfill the requirements for 95% ci according to [68]. The highest responses obtained appear in bold.

The statistical results are presented in ascending order based on the Simple Logistic responses. Table 3 is divided into two sections as follows: the first includes combinations of adj.-function words, while the second presents combinations of adverbs with function words. Table 4 displays only pairs of function words. Each table includes the results obtained from both statistical methods used in the analysis.

Overall, proximity-based evaluations demonstrate advantages over both reference groups. Specifically, for adjective–function word pairs, although the gap between proximity-based results and baseline evaluations varies, both the Naïve Bayes and Simple Logistic models show consistent performance, as the ranking of attribute combinations remains relatively stable.

For adverb–function word pairs, the combination (adv., det.) remains nearly unchanged across both baseline and proximity measures, as well as for both statistical learning methods. In contrast, the (adv., prep.) pair shows some variation in outcomes but maintains its reference position, indicating a substantial distance in evaluation compared to the other pairs in the adverbs vs. function words set. The remaining four combinations shift positions in ascending order; however, (adv., conj.) and (adv., compl.) consistently rank above (adv., aux.) and (adv., modals), respectively.

As the number of combinations increases, the relationships among function words become more dynamic. In terms of local ordering, the pairs (det., prep.) and (prep., aux.) consistently occupy the ninth and tenth positions, respectively. As before, this behavior refers to the ascending order of the directly normalized baseline, as illustrated in the graphs.

However, notable insights emerge when the data are organized in ascending order based on the proximity measure, as shown in the tables. Across the three sets—adjectives vs. function words, adverbs vs. function words (Table 3), and function words vs. function words (Table 4)—both statistical methods, Naïve Bayes and Simple Logistic, generally show consistent behavior.

The order of proximity measures between attribute pairs remains largely consistent. For example, the evaluation for the proximity measure for the pair (adj., prep.) is higher than that for (adj., conj.) for both Bayes and Logistic methods. This ranking remains consistent across most attribute combinations, with the exception of the pair (adj., det.), which deviates from the overall pattern.

The ordering of attribute pairs by ascending proximity in SimpleLogistic closely mirrors that of NaïveBayes, particularly for modifier–function word combinations, with the exception of the (adv., conj.) pair. For function word–function word pairs, seven out of ten combinations follow this consistent pattern. However, the pairs (det., modals), (compl., aux.), and (prep., aux.) deviate from the ordering observed in NaïveBayes.

## 4. Discussion

No data synthesis was performed; the analysis relied solely on a limited number of original instances. The study focused on specific linguistic attributes relevant to a narrative-discourse task and employed an alternative analytical technique. Particular attention was given to key factors influencing the problem, as their effects directly impact the study’s results [22]—and, by extension, any conclusions drawn from them.

Research indicates that cognitive training can stimulate multiple levels of cerebral function, including synaptic activity and neural network dynamics. These interventions promote neuroplasticity, leading to improvements in cognitive performance. Over time, such gains contribute to the development of more enduring cognitive foundations [69]. Contrary to expectations, the long-term functional impact of cognitive-communication disorders—common among individuals with tbi—on daily life remains under-researched [16].

Neuroplasticity-based cognitive training has been shown to mitigate the peril of a downward spiral of effects following tbi, although the evidence concerning brain plasticity specifically prompted by cognitive interventions is limited. Indeed, the rehabilitation of communication dysfunction resulting from underlying cognitive impairments—formally cognitive-communication disorder—is an emerging field [31,70].

Communication-related disability must be prioritized in rehabilitation, given the essential role communication plays in daily functioning, and consequently, long-term well-being. Previous studies have reported significant cognitive side effects [71,72,73,74] in individuals with moderate to severe tbi within the first month post-injury, and persistent cognitive impairments beyond the first three months can indicate long-term disability. Therefore, the patterns reported at three months in this study may provide relevant and meaningful insights.

Moreover, a growing body of epidemiological research has linked tbi with increased risks of age-related cognitive decline and dementia [32]. In this context, accurate information is crucial for enhancing the effectiveness of therapeutic interventions, particularly at the three-month post-injury stage. This examination provides a detailed analysis of language-related deficits, specifically identifying items that do not integrate properly within the language structure.

Function words provide essential structural cues, indicating how elements in a sentence relate to one another [75]. The marked difference in their usage between the experimental and control groups may indicate a functional language disorder or a deeper cognitive impairment.

At the beginning of the last century [76], research began to unravel the process of language in the brain, the effects on language capabilities caused by brain injury, and the outcome expected during the recovery stage.

Although extended argumentation [77] and often-debated results [78] exist, the narrow documentation [30,79] gives some insight.

While research findings may be open to speculation, the existing literature suggests the possibility of stimulating neuroplasticity through language-based interventions [46,80,81]. The neural networks of the human brain are continuously remodeled by experience [76]. Beyond neurological mechanisms, several additional factors influence treatment outcomes [82,83], and understanding “how the brain reorganizes as a response to specific training” [70] remains a fundamental question in rehabilitation.

Although narrative is considered a gold-standard task for language analysis [4,59] and is strongly associated with cognitive functions [84,85,86], narrative-based therapy is increasingly underutilized—despite its proven and vital role in daily life [2,46,87,88].

This study investigated potential changes in language use within narratives by analyzing function words, adjectives, and adverbial associations. Using the Northwestern Narrative Language Analysis (NNLA) profile as a basis, the study suggests that a latent disparity between the control and experimental groups may not be immediately apparent. However, when examined through proximity-based method, these differences become more discernible due to their stronger discriminatory capacity.

Responses were accompanied by the appropriate statistical support. Although not every combination analyzed met the minimum sample size required for an 80% ci, most achieved the statistical power necessary for a 95% ci. Notably, with the exception of the (compl.,modals) pair, which was markedly unbalanced, most combinations were relatively balanced, particularly for the purposes of proximity-based relation calculations.

Language-based therapeutic approaches that focus on analyzing parts of speech—an aspect closely linked to an individual’s cognitive stage [20,89]—may serve as effective cognitive training methods to promote cerebral activation [30]. In particular, cognitive-communication language interventions can facilitate brain reorganization mechanisms initiated by tbi, while also promoting neuroplasticity.

We examined specific language attributes and, with statistical support, identified subtle changes in language use. These findings offer valuable insights for guiding the design of more precise, language-based cognitive training programs. The goal is to reduce the risk of maladaptive changes [90] by developing balanced and more effective intervention mechanisms that are likely to promote compensatory neuroplasticity, leveraging the symbiotic relationship between brain function and language [6,91].

### Limitations

A key limitation of this approach is its reliance on standardized structured samples, which in principle provide consistency and comparability. The established transcription protocols require time and specialized knowledge.

## 5. Conclusions

In the context of clinically relevant information, such data could inform policies and practices addressing cognition-related language disorders. Using an alternative data engineering approach within the proposed framework, the study revealed specific alterations in language associated with traumatic brain injury.

The recovery of cognitive-communication competence is heavily dependent on neuroplastic brain mechanisms [1]. Identifying disrupted item-based relationships within the language structure can provide direct insight into one of the potential root causes of impairment. This, in turn, enables the formulation of more targeted interventions that aim to stimulate the brain effectively and promote neuroplasticity.

## 6. Directions for Future Research

Although only a portion of the examined pairs achieved high statistical power, the results nonetheless provide meaningful support for the continuation and expansion of research in this area. The adopted approach revealed significant differences in language use between the experimental and control groups, underscoring the method’s potential in identifying subtle linguistic variations. These findings merit further investigation across multiple dimensions. For instance, observed polarity patterns may signal underlying difficulties in sentence planning [92] or disruptions in discourse coherence [19]. However, the most immediate and necessary step is to replicate the study using a new sample and an alternative task in order to validate and extend the generalizability of the current findings.

## Figures and Tables

**Table 1:** Modifiers and function words.

Attribute	Description ^1^	Attribute	Description
‘adj.’,	# of adjectives	‘conj.’,	# of conjunctions
‘adv.’,	# of adverbs	‘complementizers’,	# of complementizers
‘det.’,	# of determiners	‘prep.’,	# of prepositions
‘aux.’,	# of auxiliaries	‘modals’,	# of modals
			and modal auxiliaries

^1^ Attributes’ formal definition are found in [64].

**Table 2:** Attribute pairs-numbers.

Attr.	Attr.	#inst.	#inst.	Attr.	Attr.	#inst.	#inst.
**1**	**2**	**Study**	**Control**	**1**	**2**	**Study**	**Control**
adj.	conj	43	41	adv.	conj	43	41
-	compl. ^1^	42	38	-	compl.	42	38
-	det.	43	42	-	det.	43	42
-	prep.	44	42	-	prep.	44	42
-	aux.	39	40	-	aux.	39	40
-	modals	42	40	-	modals	42	40
compl.	aux.	39	40	prep.	aux.	44	42
compl.	modals	38	29	compl.	det.	43	42
conj.	aux	41	42	det.	conj.	43	42
compl.	conj.	42	42	det.	prep.	43	42
modals	conj.	44	42	det.	modals	44	42

^1^ compl. = complementizers.

**Table 3:** Mod.adj./adv. vs. function-words | AUC.

	Naïve	Bayes		Simple	Logistic	
**Adj. vs.**	**Direct**	**Normalized**	**Proximity**	**Direct**	**Normalized**	**Proximity**
	**Raw-Data**	**Raw-Data**	**Relation**	**Raw-Data**	**Raw-Data**	**Relation**
compl. ^1^	0.743 ± (0.090)	0.539 ± (0.106)	**0.874** ± (0.065)	0.684 ± (0.097)	0.554 ± (0.106)	**0.927** ± (0.050)
conj.	0.729 ± (0.090)	0.598 ± (0.102)	**0.913** ± (0.053)	0.685 ± (0.095)	0.581 ± (0.102)	**0.935** ± (0.046)
prep.	0.780 ± (0.082)	0.622 ± (0.099)	**0.949** ± (0.040)	0.744 ± (0.087)	0.611 ± (0.100)	**0.952** ± (0.039)
det.	0.765 ± (0.085)	0.525 ± (0.103)	**0.942** ± (0.043)	0.736 ± (0.089)	0.572 ± (0.102)	**0.956** ± (0.038)
modals	0.740 ± (0.089)	0.575 ± (0.103)	**0.967** ± (0.033)	0.709 ± (0.092)	0.600 ± (0.101)	**0.963** ± (0.035)
aux.	0.681 ± (0.096)	0.630 ± (0.100)	**0.983** ± (0.024)	0.695 ± (0.095)	0.610 ± (0.102)	**0.982** ± (0.024)
**Adv. vs.**						
modals	0.636 ± (0.098)	0.481 ± (0.103)	**0.858** ± (0.067)	0.682 ± (0.094)	0.500 ± (0.103)	**0.885** ± (0.061)
compl.	0.629 ± (0.099)	0.499 ± (0.103)	**0.888** ± (0.060)	0.643 ± (0.098)	0.553 ± (0.102)	**0.902** ± (0.056)
aux.	0.549 ± (0.102)	0.541 ± (0.102)	**0.890** ± (0.059)	0.622 ± (0.099)	0.425 ± (0.102)	**0.907** ± (0.055)
conj	0.636 ± (0.098)	0.551 ± (0.102)	**0.887** ± (0.060)	0.614 ± (0.099)	0.457 ± (0.103)	**0.929** ± (0.048)
prep.	0.711 ± (0.091)	0.552 ± (0.102)	**0.922** ± (0.050)	0.752 ± (0.086)	0.649 ± (0.097)	**0.935** ± (0.046)
det.	0.751 ± (0.086)	0.528 ± (0.103)	**0.953** ± (0.039)	0.718 ± (0.090)	0.500 ± (0.103)	**0.957** ± (0.037)

^1^ compl. = complementizers.

**Table 4:** Function-words. vs. function-words | AUC.

	Naïve	Bayes		Simple	Logistic	
**Pair of**	**Direct**	**Normalized**	**Proximity**	**Direct**	**Normalized**	**Proximity**
**Attributes**	**Raw-Data**	**Raw-Data**	**Relation**	**Raw-Data**	**Raw-Data**	**Relation**
compl.-conj.	0.656 ± (0.098)	0.301 ± (0.094)	**0.869** ± (0.066)	0.622 ± (0.100)	0.530 ± (0.104)	**0.880** ± (0.063)
modals-conj.	0.658 ± (0.096)	0.429 ± (0.102)	**0.910** ± (0.054)	0.677 ± (0.095)	0.469 ± (0.103)	**0.894** ± (0.058)
compl.-modals	0.614 ± (0.109)	0.404 ± (0.112)	**0.931** ± (0.052)	0.720 ± (0.099)	0.492 ± (0.114)	**0.913** ± (0.058)
det.-modals	0.778 ± (0.082)	0.474 ± (0.103)	**0.915** ± (0.052)	0.725 ± (0.090)	0.466 ± (0.103)	**0.926** ± (0.049)
compl.-aux.	0.599 ± (0.105)	0.396 ± (0.105)	**0.853** ± (0.072)	0.611 ± (0.104)	0.529 ± (0.107)	**0.928** ± (0.051)
compl.-det.	0.752 ± (0.087)	0.448 ± (0.103)	**0.940** ± (0.044)	0.746 ± (0.087)	0.492 ± (0.104)	**0.932** ± (0.047)
det.-conj.	0.771 ± (0.084)	0.374 ± (0.100)	**0.942** ± (0.043)	0.738 ± (0.089)	0.378 ± (0.100)	**0.949** ± (0.041)
det.-prep.	0.773 ± (0.084)	0.548 ± (0.103)	**0.944** ± (0.043)	0.726 ± (0.090)	0.642 ± (0.098)	**0.957** ± (0.037)
prep.-aux.	0.752 ± (0.086)	0.621 ± (0.099)	**0.936** ± (0.045)	0.785 ± (0.081)	0.681 ± (0.094)	**0.959** ± (0.036)
conj.-aux	0.654 ± (0.099)	0.282 ± (0.092)	**0.969** ± (0.032)	0.668 ± (0.098)	0.406 ± (0.102)	**0.963** ± (0.035)

## Data Availability

The original contributions presented in this study are included in the article. Further inquiries can be directed to the corresponding author.
